# Purulent Ophthalmia

**Published:** 1848

**Authors:** E. G. Meek

**Affiliations:** Late of Choctaw Agency, west of Arkansas


THE NORTH-WESTERN

### MEDICAL AND SURGICAL JOURNAL.

Vol. I.] JUNE AND JULY, 1848. [No. 2.

### Part I.—Original Communications.

### ARTICLE I

*Purulent Ophthalmia.* By Dr. E. G. Meek, late of Choctaw Agency, west of Arkansas.

It is needless to go into any statistical or historical notice of this pestilent malady. A disease so rapid in its progress and destructive in its consequences, has, doubtless, been a matter of careful study with every intelligent member of the profession. The writer proposes to give such facts as came under his notice, and his deductions therefrom, during a visitation of this terrible scourge, in the winter of 1847–8. The weather, during the winter, was unusually mild and temperate; so much so that vegetation had made some progress as early as the 1st of February. The first case of ophthalmia in the neighborhood occurred about the middle of January, and the epidemic was at its highest on the 1st of March, from which time it began to decline, ceasing almost entirely, the 1st of April. The great majority of the cases were Indians. From a record of more than sixty cases, the following are presented, with the hope that they may prove interesting to the older practitioner, and instructive to those who, like the writer, have been but recently freed from their professional leading strings:

A stout lad was attacked with inflammation of the conjunctiva, a sense of heat and smarting in the eyelid, and a deep-seated pain in the orbit. On the following morning I was summoned to see him, and found the lids and integuments so much swollen as to render it difficult to obtain a view of the eye-ball; the lids were glued together, except at the inner angle, from which pus was discharged. He persisted in attributing his disease to having gotten sand in his eyes, and insisted that if this were removed he should be well. He was bled freely from the arm, his eyes cleansed with tepid water, and a solution of the nitrate of silver, of the strength of four grains to the ounce of water, was left, with directions that the eye should be washed with it once every three hours. On the following day, the severity of the symptoms was but slightly abated, and recourse was had to a second venesection; the solution being continued as before. On the third day, blisters were applied to the temples, and the patient gradually recovered, but so slowly that I had reason to be dissatisfied with the practice resorted to in his case, and resolved to alter it should opportunity occur. During his convalescence, two other lads were attacked in a similar manner; one of them in both eyes; the other in one only; both of them were bled to the verge of syncope, with immediate relief to their eyes; the strength of the caustic solution was increased tô six grains, and the patients were briskly purged with calomel and sulphate of magnesia. On the subsequent day, the severity of the symptoms was materially lessened; the venesection was not repeated; the calomel was omitted; the solution was continued as before, and the sulphate of magnesia administered at bed-time. These boys recovered more rapidly than the last mentioned, although their attack was regarded as equally violent.

The following case is detailed as presenting more points of interest than either of the foregoing. Mrs. H., an old half-breed lady, and exceedingly corpulent, had previously had an attack of ophthalmia, which resulted in total disorganization and destruction of the right eye; she having failed to apply in time for relief. On the present occasion she became alarmed, and applied for relief in good season. I found her with the integuments much swollen, palpebral conjunctiva intensely inflamed, and the sclerotica a complete net-work of injected capillaries; pus was profusely discharged; there was deep-seated pain in the orbit, and a distressing throbbing of the temple; there was but little fever or constitutional disturbance of any kind. It may be well to remark, that the appendages of the disorganized eye were not affected. Now, here was a violent case, demanding prompt and efficacious treatment, while a want of success would leave the unfortunate patient in total darkness. To put a lancet into her arm with the view of drawing blood, was going on a very uncertain voyage of discovery; for, where there was not the least trace of a vein to be seen—as was the case in her gigantic arm—venesection was an operation to be performed—with reverence be it spoken—by faith and not by sight. In this dilemma her temple was scarified, but from some cause it did not bleed freely, and two cups were applied to the back of her neck, from which nearly a quart of blood was rapidly abstracted with a remarkable relief to the patient, the pain in the eyeball being mitigated, and the engorged capillaries relieved of a great part of their contents. With some hesitation I then prepared a solution of the *argent. nitras*, of twenty grains to the ounce, and dropped a few drops in at the internal angle of the eye. The effect was to change the color of the lining membrane from vivid red to white; and that without causing any great pain to the patient, who described her sensation by remarking that her eye felt as though it had been eating green persimmons. On the following morning the solution was repeated, causing a much greater degree of pain; the eye having become sensitive, and having undergone a remarkable change for the better. On the following day the improvement still went on rapidly; the strength of the solution was reduced to ten grains, and I ceased my attendance; the subsequent recovery was prompt and complete. The satisfactory result in this case led me to the total abandonment of general remedies to combat a local disease, and, in subsequent cases, induced me to rely wholly on the scarificator and nitrate of silver, to the exclusion of the lancet, blisters, and even purgatives, except so far as to maintain a soluble condition of the bowels.

In the following case the principal point of interest is the effusion of lymph into the anterior chamber of the eye, constituting what are usually denominated nebulæ in the cornea. The subject was a gentleman who was laboring under a severe attack characterized by the symptoms before enumerated, with the addition of three nebulæ in one eye, and two in the other. He was seen early; was at once freely cupped on the nape of the neck, and put on the use of the caustic solution of the strength of fifteen grains to the ounce. In two days the effused lymph was absorbed, and the cornea perfectly clear, without further medication. In his case, as in the last, the recovery was rapid and perfect. Several cases exhibited a greater or less degree of effusion into the anterior chamber; but under prompt treatment they proved quite as manageable as those which ran the ordinary course.

One more example is presented as possessing a painful degree of interest, from the suffering occasioned to the patient, and the melancholy termination of the disease. Dr. J. P. H., a member of the Royal College of Surgeons, for many years in the service of the Hudson Bay Company in his professional capacity, came to spend the winter in our pleasant latitude, and after some weeks spent at the place of my residence, took quarters at the distance of five miles, where he was attacked in the left eye, by the prevailing epidemic. He did not seek aid, and it was some days before I knew of his situation, when, waiving all ceremony, I went at once to see him. He removed from his lodgings back to our station; but it was unfortunately too late. He was cupped to the amount of a quart, and a pencil of caustic was applied to the inflamed surface of the eyelids, in addition to the use of the solution. This produced temporary relief, but in three days the symptoms returned, accompanied with an exquisite tenderness of the scalp on the left side of his head. He was again cupped, the caustic solution continued, and took thrice in the day the following mixture:

℞.—Liq. Potass. Arsen., gtt. xv.;

Tinct. Opii, gtt. lx.

Under this treatment the disease was again mitigated; but a worse symptom now appeared; the cornea commenced to ulcerate, and, during a night, passed by the unfortunate gentleman in great agony, burst; the discharge of the aqueous humor being succeeded by an interval of ease. Suppuration then set up in the interior structures of the eye; the vitreous humor and lens were, in this way, discharged, and the coats of the eye collapsed, completing the ruin of the unfortunate organ. But all was not yet over. The inflammation of the lids still went on, with deep-seated pain referred to the collapsed eye—distressing throbbing of the temple, and tenderness of the scalp. He was again largely bled—the arsenical solution was continued—blisters were kept open in the temple and nape of the neck, and the antiphlogistic regimen strictly observed. I suggested a trial of the influence of mercury; but this was declined. He improved somewhat, but still suffers great inconvenience, from which it will probably be long before he recovers; for, so far as my observation goes (and I have seen several instances), this disease, in a chronic form, is peculiarly intractable; and should the delicate structures eventually escape destruction, it will be at the expense of protracted suffering, and permanent inconvenience to the eyelids.

Many more cases illustrative of this malady might be given, but it is hoped the foregoing may be sufficient to give a correct idea of the diagnosis, prognosis, and treatment; and it is now proposed to devote a small space to the *ratio medendi* of the course of treatment pursued in these cases. In the first place, as to the abstraction of blood. This disease is generally local, and it is believed that topical remedies are not only adequate to the emergency, but preferable to general measures, which produce inconvenience without corresponding benefit. The scarificator is, therefore, preferable to the lancet; because, in this way, blood is drawn away from the diseased organ more directly. It is true, blood may be taken from the jugular vein or temporal arteries, close to the seat of the disease; but these operations are attended with a degree of risk, which the inexperienced practitioner dislikes to assume; whereas the scarificator is, *at least*, innocuous in the hands of the veriest bungler; moreover, by dexterous management, enough blood can be taken in this way to produce the effect of a general bleeding. For the operation of cupping, the nape of the neck possesses advantages over the temple; but this is a matter of small importance.

The nitrate of silver combines two properties which render it the great remedy in ophthalmia: it is both stimulant and astringent. Modern researches into the pathology of inflammation, appear to demonstrate, that the capillaries of the inflamed organ become distended with blood, and that the distension destroys the tone of the vessels. Abstraction of blood empties them, and the nitrate of silver tends, by its twofold action, to restore their elasticity, and diminish their caliber; and the action of this remedy is a strong proof of the soundness of the prevailing theory of inflammation.

It is believed that active purgatives are unnecessary, and, therefore improper. Emetics are thought to be injurious, from their effect on the cerebral circulation. The only local application (except the caustic) should be either cold or tepid water. Poultices of all kinds are inadmissible, as keeping the eye hot, and preventing a free discharge of secreted matter. The same remark will apply to shades and bandages for excluding the light and air. Air is not injurious; and the only proper mode of excluding the light, is to darken the chamber; care being taken to increase the amount of light as fast as the eye can comfortably sustain it, lest the organ by being long darkened should become inconveniently delicate, and be thereby liable to relapse after the patient goes abroad. During the acute stage of the disease, blisters are of no service; but are no doubt valuable when the disease becomes chronic, and to derive advantage from them, they should be kept open for a length of time.

In seeking for the origin and causes of ophthalmia, authors seem to agree that it came originally from Egypt, and that the dazzling light of the sun reflected from the sandy deserts of that country, have a very deleterious influence on the eyes. One author notices an opinion current in Egypt, that the moon's rays are quite as injurious as those from the sun. It is further supposed that this is a disease of warm climates, and this supposition finds support in the fact that destructive diseases of the eye, are less common in these latitudes where the ground is covered for a great part of the year with snow; although this would seem to afford a surface of quite as great a reflective power as the deserts of lower latitudes. With us, the great prairies offer a substitute for the sand plains; and when we add that, during the most intense heat of summer, the Indian traverses these great wastes with the head bare, or enveloped in a huge woolen shawl, we have a temperature sufficiently high for anything.

Medical writers have occasionally indulged in speculations upon the color of the eyes that are most obnoxious to this disease, and have come to the conclusion that dark eyes are most frequently attacked; but this will seem a lame conclusion, when we reflect that the inhabitants of ophthalmic countries are very generally dark-eyed, and that it is difficult for any one but a lawyer to make a distinction where there is no difference. I will here, however, state a fact with reference to the color of the eyes, which may interest those curious in such matters. In a family of my acquintance there are seven children, four of whom have black eyes, and the remainder blue; the latter are short sighted, while the others exhibit no such defect.

It is a question whether or not ophthalmia be contagious. My own experience has left me but little doubt on this subject. It appears to me that there is as strong proof of the contagiousness of this disease as there is of the infectious character of small pox. A number of my patients were pupils in a public school, and the progress of the disease could be traced from chamber to chamber, and from one bed to another, and supervened promptly on the accidental application of the matters from a diseased eye. This will suggest the proper precaution to be adopted, and even should the practitioner be a non-contagionist, due precaution will be an error on the safe side.

